# Development of Gold Nanoparticle Micropatterns for the Electrical Detection of Proteins

**DOI:** 10.3390/nano11020528

**Published:** 2021-02-19

**Authors:** Geonwoo Lim, Kibeom Kim, Yuri Park, Myoung-Hwan Park

**Affiliations:** 1 Department of Convergence Science, Sahmyook University, Seoul 01795, Korea; dlarjsdn07@naver.com (G.L.); yrpark3918@gmail.com (Y.P.); 2 Convergence Research Center, Nanobiomaterials Institute, Sahmyook University, Seoul 01795, Korea; kibumsy@syu.ac.kr

**Keywords:** protein detection, imprinting, gold nanoparticles, electrical resistance, micropatterns

## Abstract

Protein analysis can be used to efficiently detect the early stages of various diseases. However, conventional protein detection platforms require expensive or complex equipment, which has been a major obstacle to their widespread application. In addition, uncertain signals from non-specific adhesion interfere with the precise interpretation of the results. To overcome these problems, the development of a technique that can detect the proteins in a simple method is needed. In this study, a platform composed of gold nanoparticles (GNPs) was fabricated through a simple imprinting method for protein detection. The corrugated surface naturally formed by the nanoparticle assemblies simultaneously increases the efficiency of adhesion and binding with analytes and reduces undesired interactions. After forming the GNP micropatterns, post-functionalization with both cationic and neutral ligands was performed on the surface to manipulate their electrostatic interaction with proteins. Upon protein binding, the change in the electrical values of the micropatterns was recorded by using a resistance meter. The resistance of the positively charged micropatterns was found to increase due to the electrostatic interaction with proteins, while no significant change in resistance was observed for the neutral micropatterns after immersion in a protein solution. Additionally, the selective adsorption of fluorescent proteins onto the micropatterns was captured using confocal microscopy. These simply imprinted GNP micropatterns are sensitive platforms that can detect various analytes by measuring the electrical resistance with portable equipment.

## 1. Introduction

Protein analysis is exceptionally promising for the detection of various diseases [1,2,3,4,5]. Notably, diagnoses of various diseases require only a small amount of the specimen. However, current conventional technologies for protein detection require labeling of target proteins or fluorophores and advanced optical imaging systems, including bulky and expensive equipment such as fluorescence and confocal microscopy [6,7,8]. These optical imaging systems have the advantage of directly observing the protein with high detection ability, but they are immobile and require significant floor area [9]. Furthermore, since additional modifications are required to detect specific proteins, complex processing is an integral part of the diagnosis, which limits the applications of the technique [10,11,12]. Moreover, non-specific binding has always been a long-standing challenge in detecting proteins [13]. These problems reduce the efficiency of early diagnosis through protein detection. Therefore, development of a versatile sensing platform that is portable and that can detect proteins without additional complex modifications is necessary [14,15,16,17,18].

**Scheme 1:**
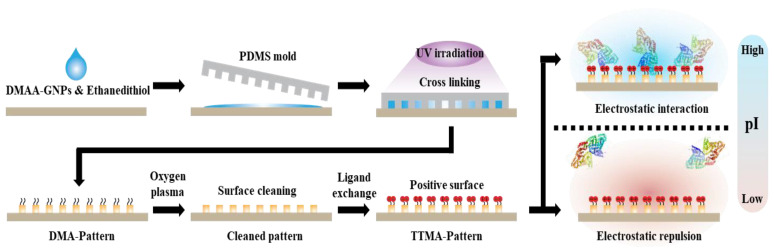
Synthesis process of a micropatterned detector and protein detection mechanism.

The microcontact imprinting technique combines microcontact printing and imprinting technologies and has been used to generate well-defined patterned surfaces for various biological analyses and biosensor fabrications [19,20,21,22,23,24,25,26]. This technique cheaply and easily generates various patterns in a limited space for the detection of small molecules, proteins, and cells and, furthermore, has been widely used to fabricate efficient platforms for sensing due to its high mechanical/chemical stability, low cost, and ease of preparation [27,28]. In this study, we developed a micropatterned platform consisting of gold nanoparticles (GNPs) that is portable and capable of detecting proteins without a complicated process. In the microchannels of the polydimethylsiloxane (PDMS) mold, the vinyl functional groups on GNPs are crosslinked with ethanedithiol upon UV irradiation, forming the nanostructured micropatterns. After simple manipulation to introduce positively charged functional groups, the micropatterns are available to detect proteins simply by measuring the resistance according to the selective attachment of the desired proteins. Proteins with negative charges in their structure at the desired pH are selectively adsorbed on the positively charged three-dimensional micropatterns by electrostatic interactions. The adsorbed proteins modify the electrical resistance of the assemblies of GNPs, thus interfering with the flow of electric current. This resistance change, measured in a simple method, can be used to detect proteins in our versatile sensing system (Scheme 1).

## 2. Materials and Methods

Citric acid and trisodium salt dehydrate (99.0%) were purchased from Samchun Pure Chemical (Gyeonggido, Korea). Sodium borohydride powder (≥ 98%), gold(III) chloride trihydrate (≥ 99.9%), triisopropylsilane (TIS; 98%), dimethylamine solution (5.6 M), 11-bromo-1-undecanol (≥ 99.0%), methanesulfonyl chloride (≥ 99.7%), allyl bromide (99%), albumin-fluorescein isothiocyanate conjugate protein bovine (FITC-Albumin), acetic acid glacial (≥ 99.0%), (3-mercaptopropyl)trimethoxysilane (95%), 1,2-ethanedithiol (≥ 98.0%), and poly(ethylene glycol) methyl ether thiol (Mn 6000) were purchased from Sigma Aldrich (St. Louis, MO, USA). Triethylamine (TEA; >99%), trifluroacetic acid (TFA; > 99.0%), and albumin from dried egg white (crude) were purchased from TCI (Tokyo, Japan). L-Ascorbic acid sodium salt (99%) and triphenylmethyl mercaptan (98%) were purchased from Alfa Aesar (Tewksbury, MA, USA). Hexadecyltrimethylammonium bromide (CTAB, 99%) was purchased from Acros Organics (Morris Plains, MA, USA). The synthesis of the dimethyl allyl amine (DMAA) ligand is reported in the Supporting information. Tetra ethylene glycol amine (TTMA) ligands were synthesized according to a reported procedure [29].

Nuclear magnetic resonance spectroscopy (NMR; Tokyo, Japan, JEOL ECX-400) was employed to characterize the DMAA and TTMA. The diameter of GNPs was confirmed by transmission electron microscopy (TEM; Tokyo, Japan, JEOL JEM-2010), dynamic light scattering (DLS; Worcestershire, UK, Malvern Instruments ZEN 3600) as an aqueous state, and optical properties of materials were observed from 400 nm to 110 0nm by ultraviolet visible spectroscopy (UV-vis; Seoul, Korea SCINCO NEOSYS-2000). Morphology of patterns was observed by scanning electron microscopy (SEM; Tokyo, Japan, JEOL JSM-6510) operating at 20 keV. Before modifying the TTMA and polyethylene glycol (PEG) ligands, patterns were cleaned by the oxygen plasma surface etching (Ebhausen, Germany, Diener Electronic ZEPTO) for 2 min at the oxygen atmosphere. Roughness of patterns was observed by atomic force microscopy (AFM; Gyeonggido, Korea, PSIA Inc. XE-100). The contact angle analyzer (Gyeonggido, Korea, SEO Phoenix150) was employed to check the contact angle of water on the pattern surface. To obtain a fluorescence image, FITC-Albumin was excited by 488 nm laser, and emission (525 nm~ 540 nm) was observed by confocal microscopy (Solms, Germany, Leica TCS SP8). Resistance was measured by 4-point probes (Gyeonggido, Korea, MS TECH M4P 302-System).

### 2.1. Synthesis of Vinyl Functionalized GNPs (DMAA-GNPs)

A solution of 0.5 mL of gold(III) chloride (HAuCl_4_, 10 mM) and 0.5 mL of trisodium citric acid (0.1 M) was added to 10 mL of deionized (DI) water to prepare the growing solution. Sodium borohydride solution (0.6 mL) was added to the above solution while stirring at 25 °C for 2 h. The seed solution was used within 2–5 h of preparation. Additionally, 1.2 g of CTAB and 10 mL of the HAuCl_4_ solution (10 mM) were dissociated in 10 mL of DI water. Ascorbic acid (0.1 M, 0.5 mL) was added to 9 mL of the resulting solution at 37 °C. The prepared seed solution was added after the solution turned colorless. The resulting solution was further stirred for 30 min. The synthesized AuNPs were centrifuged. For ligand exchange, an excess of DMAA ligands was added to the GNP solution and further stirred for 2 days. The reaction solution was centrifuged, and the GNPs were washed with ether several times and dried. 

### 2.2. Fabrication of Micropatterns and Post-Functionalization

A cleaned wafer was initially etched by oxygen plasma for 2 min, and the oxidized wafer was immediately dipped in a (3-mercaptopropyl) trimethoxysilane solution (2 wt%) for 7 h. A mixture containing 1.4 mg of DMAA-GNPs and 4 μL of an ethane dithiol solution (1 wt% in methanol) was deposited dropwise onto the thiol-modified surface, and then a PDMS mold with micropatterns was softly pressed on the surface. UV light was irradiated onto the micropatterns (GNPs inside PDMS) for 2 h to induce crosslinking by the thiol-ene reaction. The fabricated micropatterns were washed by immersion in methanol and etched by oxygen plasma to clean the surface. After etching, the micropatterns were immersed in the TTMA or PEG ligand solution (0.1 wt% in DI) for 12 h. The ligand-modified micropatterns were immersed in methanol to remove the unmodified ligand (three times).

### 2.3. Detection of Proteins by Micropatterns

Prepared patterns (1 cm^2^) were immersed in albumin or hemoglobin solution (6 mg/mL in buffer solution). After immersion, patterns were washed with DI water several times to remove extra proteins and dried. The resistance of patterns was measured by a 4-point probe. Experiments were repeated at different pH, which was controlled by a buffer solution.

## 3. Results

### 3.1. Fabrication of Crosslinkable GNP-DMAA

**Figure 1:**
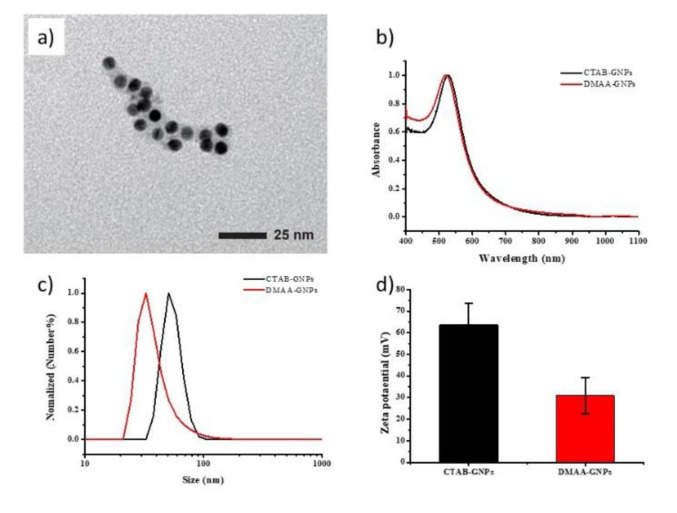
(**a**) TEM image of DMAA-gold nanoparticles (GNPs). (**b**) UV-vis spectrum, (**c**) DLS, and (**d**) Zeta potential data of CTAB- and DMAA-modified GNPs.

Assembled GNPs, which are conductive and biocompatible, were used to provide a nanostructured surface in order to enhance contact efficiency with the desired proteins and to enable sensitive transmission of signals upon protein attachment [30,31,32]. Initially, GNPs (8 nm in diameter) with CTAB attached were prepared using a seed growth method [33], and CTAB-GNPs were further functionalized with DMAA ligands through a common ligand exchange method [34,35,36]. The successful exchange of ligands onto the GNPs was confirmed using TEM, UV-vis, DLS, and zeta potential measurements. Initially, the TEM image confirmed that the sphere shape of GNPs was retained after the ligand exchange from CTAB to DMAA (Figure 1a) and no meaningful change was observed in the optical properties by UV-vis measurements (Figure 1b). The DLS analysis showed significantly larger coated particles than the solid particles (approx. 8 nm) investigated by TEM. The increased characteristic values are 54 ± 10 nm and 25 ± 13 nm for CTAB-GNPs and DMAA-GNPs, respectively. The general increase in the size of GNPs is the consequence of the physical principle of DLS measurement. Namely, the measured hydrodynamic diameters involve the whole interface regions containing the DMMA/CTAB molecules in different states and the extra water shells of the outer regions. The loose electrostatically coated CTAB on a GNP results in a more extended interfacial region, and consequently higher diameter than that of GNPs coated by DMMA with tight covalent bonds. These differences, in the interaction between the ligands and particles, and the alteration, in the length of ligand molecules, cause a huge increase of the CTAB-GNP diameter on DLS measurement, and the difference in diameter means the successful ligand exchange from CTAB to DMMA. In addition, the exchange of ligands from CTAB to DMAA induces a greater density of positive charges, resulting in a ζ potential decrease from +63.7 mV to +30.9 mV (Figure 1d).

### 3.2. Fabrication of GNP Micropatterns and Post-Functionalization

**Figure 2:**
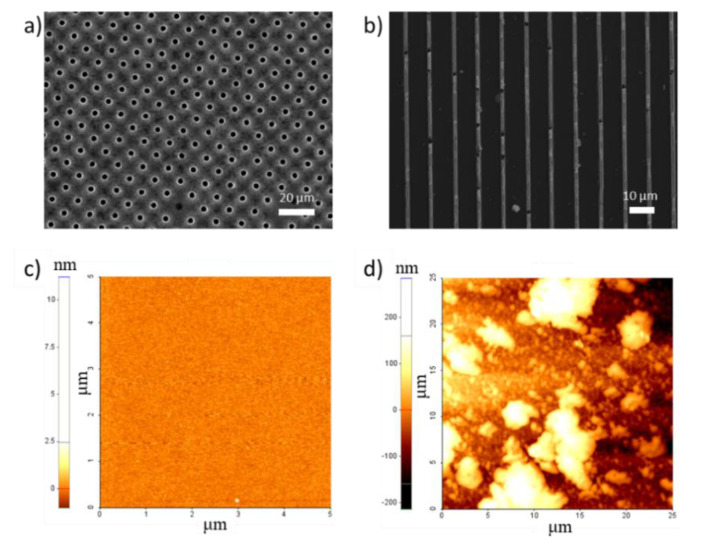
SEM images of (**a**) hole and (**b**) line micropatterns. AFM images of a (**c**) bare silicon wafer and (**d**) DMAA-GNP-imprinted wafer.

A microcontact imprinting method was used to fabricate the GNP micropatterns. A solution containing DMAA-GNPs and ethanethiol was dropped onto the thiol-functionalized substrate, and a PDMS mold was lightly placed on the substrate using low pressure. To form the micropatterns, a thiol-ene reaction of the vinyl functional groups of DMAA-GNPs with the thiol groups of ethanedithiol was induced upon UV irradiation, crosslinking each GNP. This contact imprinting-mediated method is used to fabricate various platforms by using a well-designed PDMS mold without the need for expensive or complex equipment. The fabricated hole and line micropatterns were validated by SEM analysis. For the hole patterns, the diameter was 2.5 μm and the distance between each hole was 10 μm (Figure 2a), and for the line patterns, the line width was 2.3 μm and the distance between each line was 3.8 μm (Figure 2b).

In addition, the nanostructured surfaces naturally formed by the nanoparticle assemblies can enhance specific binding but reduce undesired interactions, as reported in the literature [37,38]. As shown in Figure 2c,d, AFM revealed a smooth surface with no difference in height for the bare gold substrate, while an uneven surface was observed on our micropatterns with high roughness due to the irregular coagulation of GNPs after crosslinking by a thiol-ene reaction. High roughness indicates an increase in surface area, which blocks non-specific binding and induces interactions with the desired proteins along with the pattern effect for protein detection.

**Figure 3:**
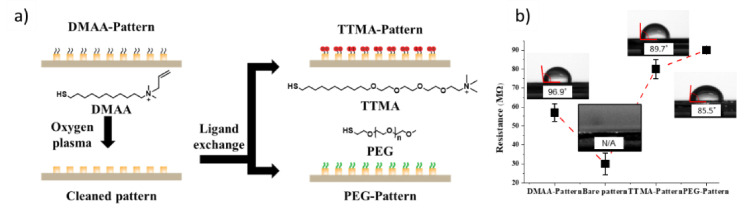
(**a**) Fabrication procedure of TTMA- and PEG-patterns. (**b**) Resistance and contact angle changes of patterned surfaces.

To improve the binding with proteins, TTMA thiol ligands with quaternary ammonium and thiol functional groups in the structure were introduced onto the micropatterns after activating the GNP surface through oxygen plasma etching (hereafter denoted as TTMA-patterns). Under the same conditions, the cleaned micropatterns were modified with neutral PEG thiol ligands by Au-thiol bonds (hereafter denoted as PEG-patterns) (Figure 3a). PEG, which endows high biocompatibility and has low interactions with various biomaterials, is widely used to prevent unintended adhesion in medical applications [39,40,41]. The TTMA and PEG ligand modification was confirmed using SEM and contact angle and resistance measurements. In the SEM image, damage or morphological change was not observed even after ligand modification (Figure S1). The contact angles of the functionalized surfaces, including the DMAA and etched wafers and the TTMA- and PEG-pattern, are 96.88°, n/a, 89.1°, and 85.5°, respectively (Figure 3b). Functionalized GNPs on the micropatterns allow the movement of electrons, while GNPs with reduced conductivity interrupt the current flow and affect the resistance of the micropatterns. Therefore, both post-modification and protein adsorption can be confirmed by measuring the resistance. Initially, 57.72 MΩ was measured for the DMAA patterns, and this value decreased to 30.89 MΩ after oxygen plasma etching, which then increased upon further treatment with TTMA (80.03 MΩ) or PEG ligands (90.00 MΩ) (Figure 3b). These results indicate that the micropatterns were successfully modified with the TTMA and PEG ligands.

### 3.3. Detection of Proteins on GNP Micropatterns

**Figure 4:**
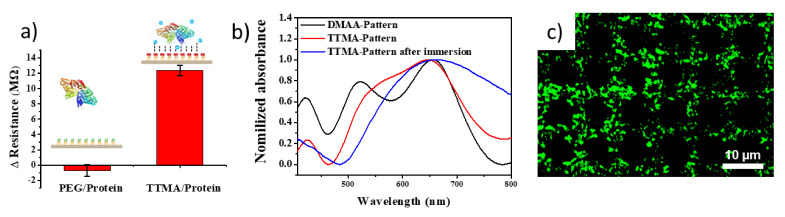
(**a**) Resistance changes in TTMA-pattern and PEG-pattern after immersion in albumin solution. (**b**) UV-vis data for DMAA, TTMA-pattern, and TTMA-pattern after immersion in albumin solution. (**c**) Confocal image of TTMA-pattern after immersion in FITC-albumin solution.

The conductive GNP micropatterns enable the movement of electrons, and the sensitivity of the micropatterns is increased by the adsorption of positively charged substances to facilitate the detection of proteins by changes in resistance. The prepared TTMA-pattern showed a significant increase in resistance of approximately 12.3 MΩ after immersion in an albumin solution, while no significant change was observed for the PEG-patterns that have low interaction with proteins due to the protecting feature of PEG-patterns for non-specific binding with biomolecules (Figure 4a). These resistance changes suggest that electrostatic interactions are the main driving force for protein adsorption and desired proteins can be detected with our TTMA-pattern by measuring the resistance. In addition, the TTMA-pattern was fabricated on a transparent slide glass instead of a nontransparent silicon wafer to observe the changes in optical properties using UV-vis spectroscopy after the ligand change and protein adsorption. After TTMA post-functionalization, the absorbance peak was broadened due to the higher charge density of TTMA than of DMAA. The TTMA-pattern can selectively interact with albumin to induce a red shift in absorbance after immersion in an albumin solution (Figure 4b). As reported before, optical characteristics of GNPs become wider and red-shift after the formation of dry composites because GNPs are very close to each other but retain their optical properties as nanoparticles [42,43]. The selective binding of albumin was further confirmed using fluorescent albumin (FITC-albumin), providing a green confocal image corresponding with the TTMA-pattern (Figure 4c).

**Figure 5:**
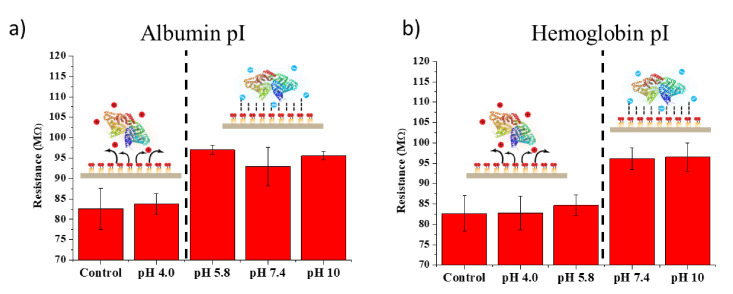
Resistance changes in TTMA-pattern after immersion in various pH solutions of (**a**) albumin and (**b**) hemoglobin.

Interestingly, the TTMA-pattern showed pH-dependent protein detection at the isoelectric point (pI) of each protein. Proteins exhibit a positive surface charge below the pI, but a negative surface charge above the pI in a solution. In fact, a significant change in resistance was not observed after immersion in an albumin (pI = 4.5) solution of pH = 4.0, while the resistance value rapidly increased when the TTMA-pattern was immersed in an albumin solution of pH > 5.8, as shown in Figure 5a. This phenomenon was further confirmed using hemoglobin (pI = 6.8) that has a higher pI value than albumin. As expected, the resistance of the TTMA-pattern markedly increased after immersion in a hemoglobin solution of pH > 7.4, but no change was observed after immersion in the hemoglobin solution of pH < 5.8 (Figure 5b). These results suggest that our functionalized GNP micropatterns can be excellent platforms for the selective detection of the desired proteins according to the pI value.

## 4. Conclusions

Early diagnosis is necessary for effective and express disease treatment. Since proteins are important indicators of various diseases, most early diagnosis devices are based on protein-sensing platforms. However, most conventional methods require expensive and bulky detection equipment that is too complex to use frequently and commonly. Moreover, specific protein detection methods are needed to increase the accuracy of diagnosis. To overcome these limitations, in this study, micropatterns of crosslinked GNP assemblies were fabricated using a microcontact imprinting technique. The TTMA ligands post-functionalized on the micropatterns are positively charged, which electrostatically interact with the negative charges of proteins. The positively charged micropatterns sensitively respond to the desired proteins, thereby causing a change in resistance. Furthermore, the micropatterns selectively detect the protein according to the pI of each protein; thus, the micropatterns have the potential to distinguish between various diseases by selective protein detection. These characteristics of our versatile system facilitate effective treatment by enhancing the accuracy of early disease diagnosis. As early diagnosis platforms, GNP micropatterns are sensitive and easy to fabricate, thus facilitating miniaturization and portable applications for protein detection to assist in the prevention of serious medical conditions.

## Data Availability

The data presented in this study are available on request from the corresponding author. The data are not publicly available due to institutional policies.

## Supplementary Materials

The following are available online at https://www.mdpi.com/2079-4991/11/2/528/s1, Scheme S1: Synthesis scheme of DMAA ligand. Figure S1: SEM image of (a) DMAA-pattern, (b) TTMA-pattern, and (c) hole of TTMA-pattern. Figure S2: Resistance change of PEG-pattern after immersion in various pH solutions of albumin.
