# Supplementary material for: Development of Gold Nanoparticle Micropatterns for the Electrical Detection of Proteins
Source: Nanomaterials (Basel). 2021 Feb 19;11(2):528. doi: 10.3390/nano11020528 (PMC7922899; doi:10.3390/nano11020528)
Supplement: Supplementary file 1 [file nanomaterials-11-00528-s001.pdf]

nanomaterials

Supporting information for:

Development of Gold Nanoparticle Micropatterns for the Electrical Detection of Proteins

Geonwoo Lim[1,‡], Kibeom Kim[2,‡], Yuri Park[1], and Myoung-Hwan Park[1, 2*]

[1] Department of Convergence Science, Sahmyook University, Seoul, 01795, South Korea
[2] Convergence Research Center, Nanobiomaterials Institute, Sahmyook University, Seoul, 01795, South Korea.
‡ These authors contributed equally to the work
* Correspondence: M. H. Park (mpark@syc.ac.kr)

Scheme S1. **Scheme of synthesis of DMAA ligand.**

Figure S1. **SEM images of a) DMAA-pattern, b) TTMA-pattern, and c) hole in the TTMA-pattern.**

Figure S2. **Resistance changes of PEG-pattern after immersion in various pH solutions of albumin.**

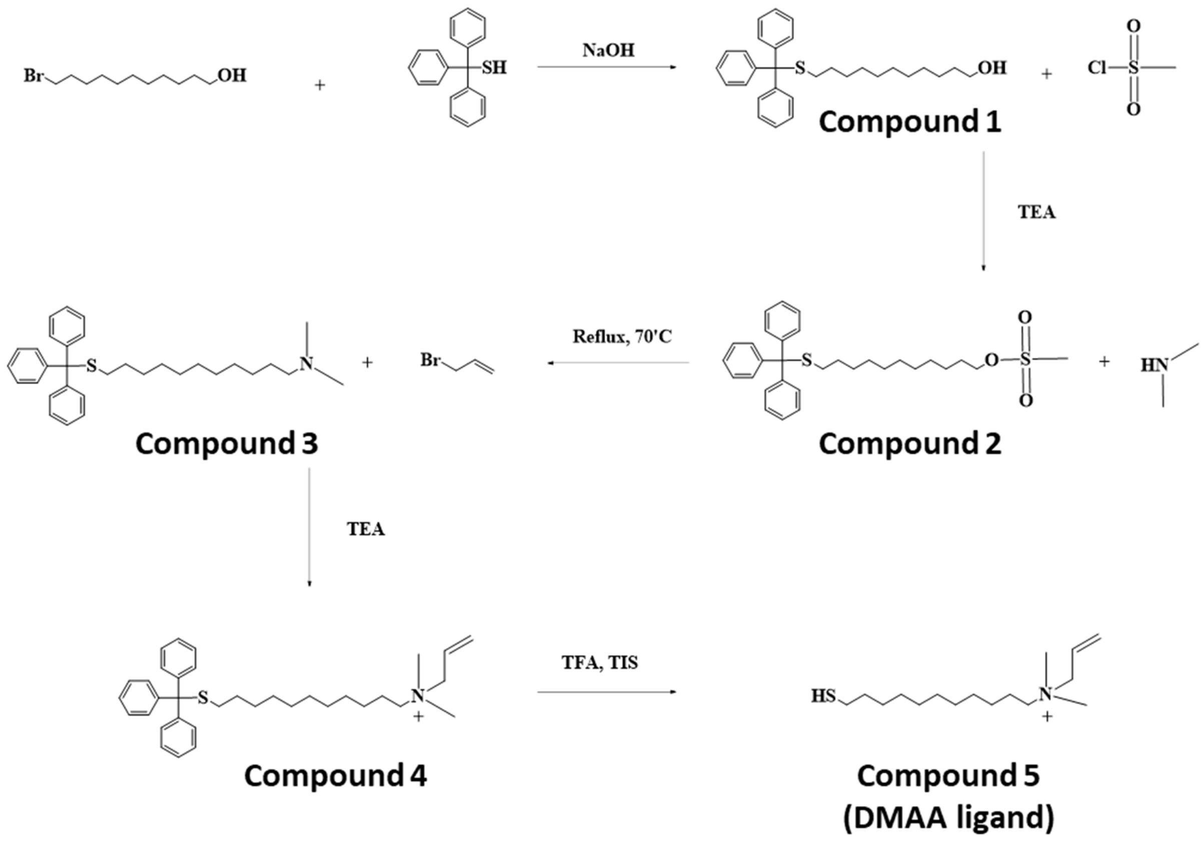

Scheme S1. Scheme of synthesis of DMAA ligand.

Synthesis of DMAA ligand

Compound 1: Triphenylmethyl mercaptan (1.65 g) was added to 10 mL of an ethanol and toluene solution (1:1 volume ratio). A saturated sodium hydroxide solution (7 mL) was added to the triphenylmethyl mercaptan solution while stirring. The resulting solution was mixed with 4 mL of a 11-bromodecanol solution (1.6 M) for 4 h with reflux at 80 °C. The product was purified using a column and confirmed by NMR.

Compound 2: A quantity of 1 g of compound 1 was dissolved in 7 mL of DCM to which TEA (0.624 mL) was added. Methanesulfonyl chloride (1.15 mL) was added dropwise to the resulting solution while stirring for 4 h. The product was purified using a column and confirmed by NMR.

Compound 3: A quantity of 1 g of compound 2 and 5.13 mL of dimethylamine was dissolved in 20 mL of ethanol. Then, 7 mL of DCM and TEA (0.624 mL) was added to a solution of compound 2. The product was purified using a column and confirmed by NMR.

Compound 4: A quantity of 1 g of compound 3 and 0.6 mL of TEA was dissolved in 30 mL of DCM while stirring for 30 min. The resulting solution was added to 10.2 mL of allyl bromide and stirred overnight. The product was purified using a column and confirmed by NMR.

Compound 5 (DMAA ligand): The TFA solution was prepared by dissolving 9.6 mL of TFA in 30 mL of DCM, and the TIS solution was prepared by dissolving 19 mL of TIS in 60 mL of DCM. A quantity of 1 g of compound 4 was dissolved in 40 mL of the TFA solution and stirred until the color changed to green from yellow. The resulting solution was added to 60 mL of the TIS solution and stirred overnight. The product was purified using a column and confirmed by NMR.

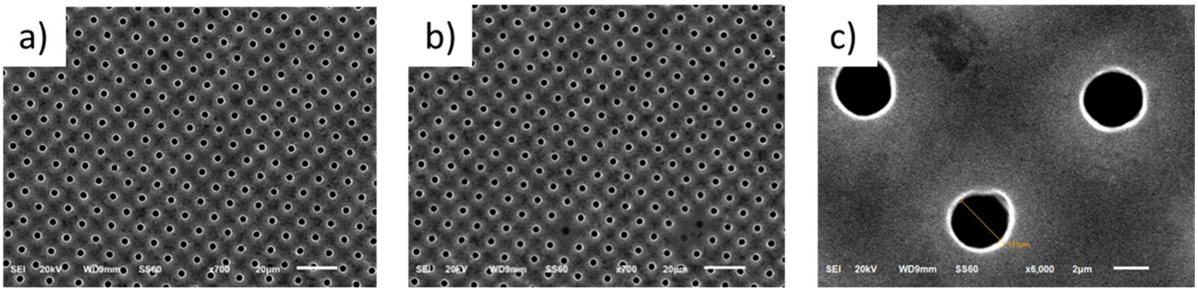

Figure S1. SEM images of a) DMAA-pattern, b) TTMA-pattern, and c) hole in the TTMA-pattern.

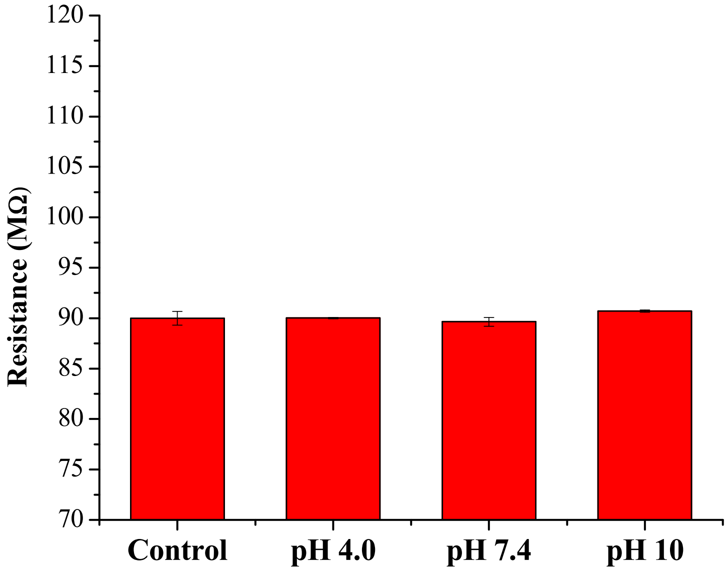

Figure S2. Resistance changes of PEG-pattern after immersion in various pH solutions of albumin.
